# DSPONVNet: a multimodal deep learning model integrating intraoperative monitoring and clinical features for predicting postoperative nausea and vomiting risk

**DOI:** 10.1186/s12874-026-02845-w

**Published:** 2026-04-02

**Authors:** Lixin Liu, Haifeng Wang, Yi Wei, Di Kong, Zhaoping Xue, Ying Liu

**Affiliations:** 1 Post-Anesthesia Care Unit, The First Hospital of Jilin University, No. 71 Xinmin, Changchun, Jilin Province 130021 China; 2 Department of Spinal Surgery, The First Hospital of Jilin University, No. 71 Xinmin, Changchun, Jilin Province 130021 China; 3 Department of Urology Surgery, The First Hospital of Jilin University, No. 71 Xinmin, Changchun, Jilin Province 130021 China

**Keywords:** Postoperative nausea and vomiting, Deep learning, Multimodal fusion, Intraoperative monitoring

## Abstract

**Purpose:**

Postoperative nausea and vomiting (PONV) is a frequent and distressing complication that affects patient comfort and recovery following surgery. Conventional risk prediction models rely heavily on static clinical features, often overlooking real-time physiological signals. This study aimed to develop a robust prediction model that integrates intraoperative monitoring data with structured clinical variables to enhance the accuracy of PONV risk assessment.

**Methods:**

We proposed DSPONVNet, a multimodal deep learning model incorporating a multilayer perceptron (MLP) for static features, a long short-term memory (LSTM) network for dynamic intraoperative monitoring data, and a self-attention mechanism for feature fusion. A total of 53,250 patients who underwent general anesthesia were retrospectively included. The model was trained and evaluated using stratified data partitioning and compared with five baseline models.

**Results:**

DSPONVNet achieved superior performance, with a ROC-AUC of 0.9376 and F1 score of 0.8701, outperforming all baseline models. SHAP analysis revealed that both traditional risk factors (e.g., female sex, prior PONV) and intraoperative heart rate fluctuation significantly contributed to risk prediction, highlighting the value of integrating dynamic physiological data.

**Conclusion:**

DSPONVNet demonstrates enhanced predictive capability and interpretability by incorporating intraoperative monitoring data, enabling more accurate and individualized PONV risk assessment. These findings support the use of real-time data fusion in clinical decision support systems for perioperative care optimization.

## Introduction

Postoperative nausea and vomiting (PONV) is a prevalent and distressing complication during the peri-anesthetic period, with an incidence ranging from 30% to 50% among surgical patients and reaching up to 80% in high-risk populations [1–3]. PONV not only compromises patient comfort but also increases the risks of dehydration, malnutrition, elevated intra-abdominal pressure, and other severe complications. These adverse outcomes contribute to delayed postoperative recovery, increased demand for medical interventions, and higher hospitalization costs [4–6]. Therefore, accurately predicting the risk of PONV is critical for enhancing the precision and timeliness of postoperative interventions, identifying key etiological factors, and optimizing perioperative decision-making and resource allocation.

Traditional approaches to predicting postoperative nausea and vomiting (PONV) were mainly based on linear logistic regression models, such as the well-known Apfel [7] and Koivuranta scores [8]. Derived from relatively small datasets and a few easily obtainable clinical variables, these models achieved simplicity, transparency, and strong clinical usability, forming the basis of current PONV risk assessment. Following this framework, Choi et al. [9] adapted the model for Korean surgical patients, Kranke et al. [10] developed and prospectively evaluated the POVOC score, a pediatric-specific risk model for predicting postoperative vomiting in children, Tan [11] proposed an obstetric model for cesarean delivery, and Chae [12] expanded the approach to a large cohort using dynamic logistic estimation. Despite these refinements, such regression-based methods remain limited by their linear assumptions, small-sample origins, and reliance on prior variable selection, achieving only moderate discriminative performance with reported AUC values typically ranging from 0.65 to 0.73.

With the increasing availability of perioperative data and advances in computational techniques, machine learning (ML) methods have been increasingly been adopted for PONV prediction. Recent studies have reported promising results using diverse ML techniques. Xie et al. [13] applied random forest, SVM, and XGBoost models to intravenous patient-controlled analgesia data, achieving AUCs of 0.95 and 0.82 in internal and external validation, respectively. Zhou et al. [3] compared multiple architectures, including AdaBoost and LightGBM, with average AUCs around 0.73. Hoshijima et al. [14] used an explainable LightGBM framework (AUC $\approx$ 0.87), while Glebov et al. [15] employed an ensemble ML model to predict early and delayed PONV with accuracies of 83.6% and 74.8%, respectively. Compared with traditional linear regression models, ML algorithms can capture complex, nonlinear relationships among perioperative variables and handle high-dimensional data without relying on strict model assumptions or manual feature selection.

Recent studies have further emphasized the importance of incorporating such dynamic signals, demonstrating that intraoperative time-series physiological data can substantially enhance the prediction of postoperative complications. Datta et al. [16] developed the MySurgeryRisk platform that integrates continuous intraoperative hemodynamic data to predict multiple postoperative adverse events, achieving substantially higher accuracy than static-feature models. Shickel et al. [17] utilized deep temporal convolutional networks on high-frequency intraoperative signals to identify patients at risk for critical postoperative deterioration. Similarly, Zhao et al. [18] applied recurrent neural networks (RNNs) to perioperative physiological trajectories to estimate recovery quality.

To date, most existing PONV prediction models remain predominantly reliant on static clinical variables, such as age, sex, American Society of Anesthesiologists (ASA) physical status, and anesthesia modality, while paying limited attention to dynamic intraoperative physiological monitoring data, including heart rate, blood pressure, and oxygen saturation. To overcome these limitations, we propose DSPONVNet, a multimodal deep learning model specifically designed for PONV risk prediction, which effectively integrates intraoperative dynamic monitoring data with structured clinical variables, enabling the simultaneous modeling of temporal dependencies within dynamic features and nonlinear interactions among static variables. This approach not only improves prediction accuracy and generalizability but also provides reliable data-driven support for individualized perioperative clinical interventions. Furthermore, this study provides supporting evidence for the application of continuous intraoperative monitoring data in postoperative complication risk prediction.

## Methods

**Fig. 1:**
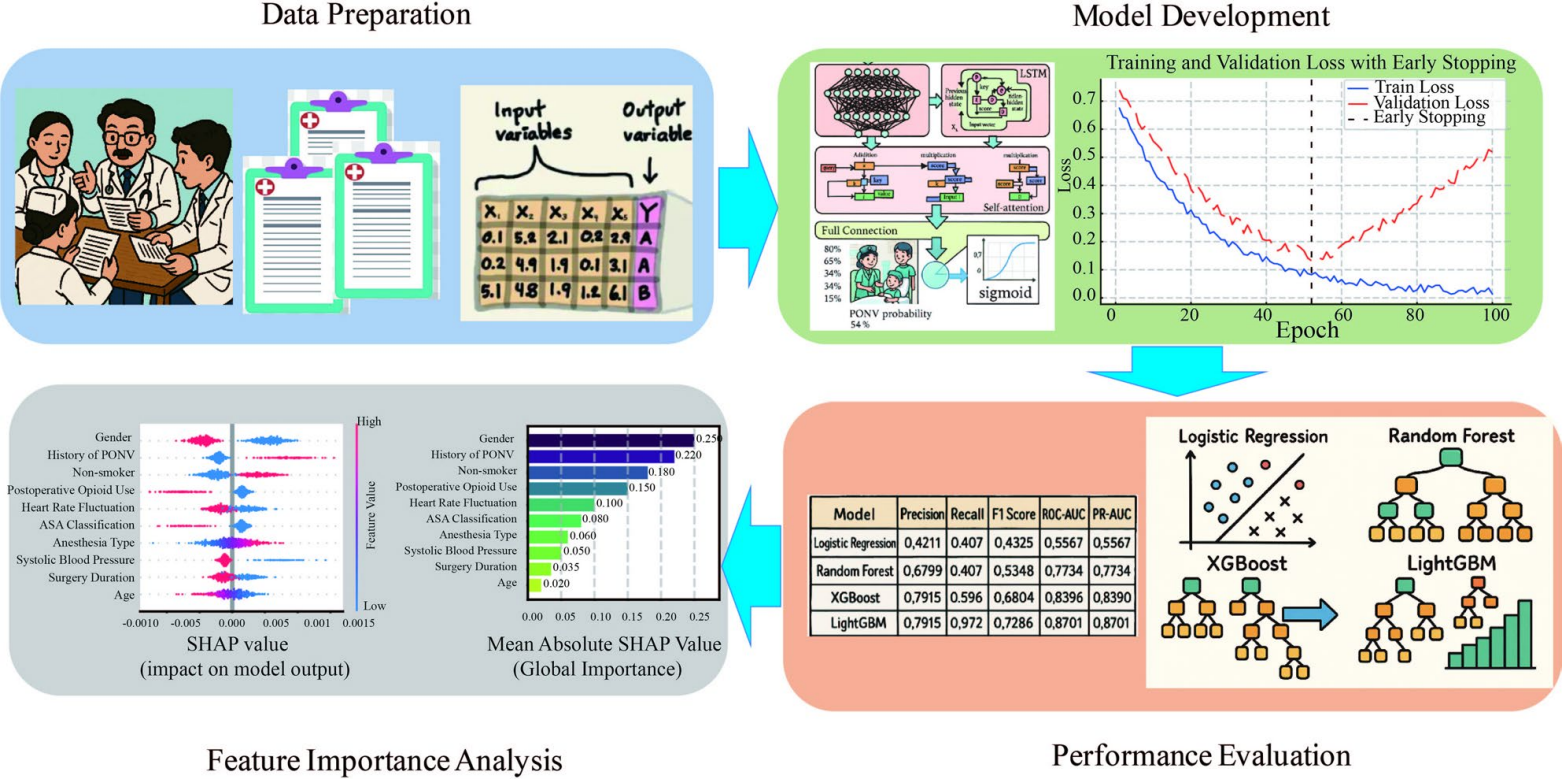
Overview of the study workflow

This section provides a comprehensive description of the methodology adopted in this study, including data preparation, model development, performance evaluation, and feature importance analysis. These components collectively form the foundation for the proposed predictive framework. The overall workflow of the study is illustrated in Fig. 1.

### Data preparation

#### Study design and ethical approval

This study was conducted as a retrospective observational analysis using electronic medical records from the First Hospital of Jilin University during the year 2024. Data were collected from adult patients who underwent general anesthesia procedures. All patient information was fully anonymized prior to analysis to ensure confidentiality. The study protocol was reviewed and approved by the Ethics Committee of the First Hospital of Jilin University (approval number: 2024-MS-117), in accordance with the Declaration of Helsinki and national regulations on biomedical research involving human participants. Given the retrospective nature of the study and the use of anonymized data, the requirement for informed consent was waived by the ethics committee.

#### Outcome definition

The primary outcome was postoperative nausea and vomiting (PONV), defined as any documented event of nausea or vomiting occurring within 24 hours after surgery, starting from the patient’s arrival at the post-anesthesia care unit (PACU). Outcome ascertainment was based on nursing records, electronic medical documentation, and medication administration logs.

#### Candidate variable selection

This study adopted an evidence-based nursing methodology and followed the “6S” hierarchical evidence model to systematically retrieve relevant literature on postoperative nausea and vomiting (PONV) risk factors from both domestic and international sources. Literature screening and quality appraisal were independently performed by two researchers, and any discrepancies were resolved through consultation with a third reviewer. Based on this process, 19 candidate predictors were identified, including 15 structured clinical features (e.g., patient demographics, surgical and anesthetic details, perioperative medication) and 4 intraoperative monitoring variables (oxygen saturation, systolic blood pressure, diastolic blood pressure, and heart rate during the anesthesia maintenance phase). These features were selected to comprehensively reflect patients’ perioperative physiological states and support robust modeling of PONV risk.

#### Patient inclusion and exclusion criteria

Clinical data were obtained from patients who underwent general anesthesia at the First Hospital of Jilin University throughout 2024, totaling 63,520 surgical cases. Based on the selected candidate variables and predefined criteria, eligible cases were screened as follows:

Inclusion criteria: (1) Age $\ge$ 18 years; (2) ASA physical status I–III; (3) General anesthesia with mechanical ventilation; (4) Surgery duration > 40 minutes; (5) Transfer to the post-anesthesia care unit (PACU) for recovery; Exclusion criteria: (1) Records with insufficient data quality, defined as cases with >30% missing baseline variables or implausible/erroneous entries (e.g., negative durations, biologically impossible values, or inconsistent timestamps); (2) Cases in which the PONV outcome could not be assessed within 24 hours after PACU admission due to postoperative events such as death, transfer, or treatment discontinuation.

Finally, 53,250 patients were included in the subsequent model development. The list of candidate features and cohort characteristics is summarized in Table 1. For categorical variables, data are shown as n (%), and for continuous variables as mean ± SD. Numbers in brackets indicate percentages, values after the ± sign represent standard deviations, and “–” denotes not applicable (e.g., numeric variables without categorical levels).

**Table 1:**
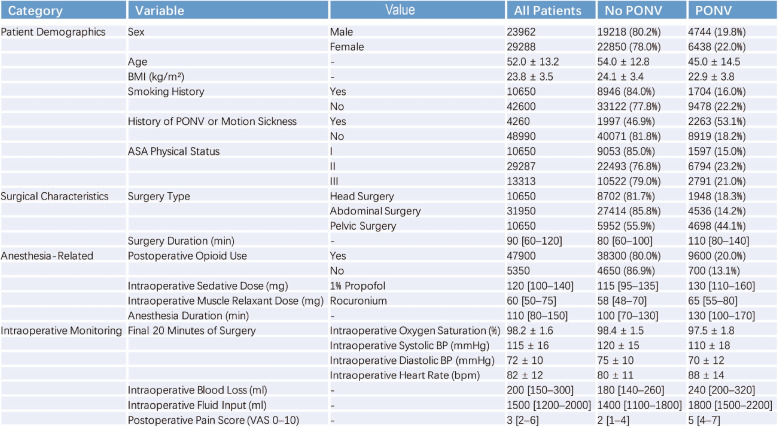
Summary of baseline characteristics

#### Data preprocessing

To improve data quality and meet the model input requirements, a series of preprocessing procedures were applied to the raw clinical dataset. These included missing value imputation, outlier correction, class imbalance adjustment, and feature normalization and encoding. The main steps are outlined as follows:

- *Handling of Missing and Erroneous Data*. Missing values and implausible entries caused by data-entry errors or recording artifacts were treated in a unified manner. Intraoperative monitoring data occasionally exhibited missing or invalid readings due to recording interruptions or equipment failures; these time-series gaps were imputed using forward-filling methods to preserve temporal continuity. For continuous clinical variables (e.g., BMI, surgery duration), missing or erroneous values were replaced with the population mean. For binary history-related variables (e.g., smoking status, prior PONV or motion sickness), missing or invalid states were retained as an additional category to capture uncertainty and potential information gain. These missing or erroneous entries accounted for less than 0.5% of all observations and had negligible impact on the modeling process.
- *Class Imbalance Processing*. Class Imbalance Processing. Due to the original PONV incidence rate of only 21%, the dataset exhibited significant class imbalance, which could adversely affect model performance on the minority (positive) class. To mitigate this, we applied a hybrid resampling strategy combining the Synthetic Minority Oversampling Technique (SMOTE) with Edited Nearest Neighbors (ENN) ,restricted to the training set only. This approach synthetically increased minority class samples while simultaneously removing noisy or borderline examples. After resampling, the positive class proportion increased to approximately 42%. The validation and test sets retained their original class distributions to ensure unbiased evaluation of model performance.
- *Feature Encoding and Normalization*. To standardize input representations, all features were encoded and normalized based on their data types. Binary variables were encoded as 0–1 values. Categorical variables with more than two levels (e.g., surgery type, ASA classification) were one-hot encoded to prevent ordinal misinterpretation. ASA classification, although ordinal in nature, was encoded using one-hot encoding to reflect its mutually exclusive categories and to avoid spurious linear relationships. Multi-label features (e.g., comorbidity history) were multi-hot encoded. All continuous variables were normalized using Z-score standardization (zero mean, unit variance) to ensure comparability across different scales and to enhance convergence efficiency during model training.

After preprocessing, the final dataset included 63 features, comprising 23 structured clinical variables and 40 time-series features derived from intraoperative monitoring. Specifically, four physiological parameters—heart rate, oxygen saturation, systolic blood pressure, and diastolic blood pressure—were continuously recorded every 2 minutes during the final 20 minutes of surgery, yielding 10 consecutive values per parameter. These measurements were preserved in sequential order to maintain temporal patterns. All preprocessing procedures were implemented using Python 3.9.13, with commonly used libraries including Pandas (v1.5.3), Scikit-learn (v1.2.2), Imbalanced-learn (v0.10.1), and NumPy (v1.23.5). Details on feature categories, encoding methods, and dimensional breakdowns are provided in Table 2.

**Table 2:**
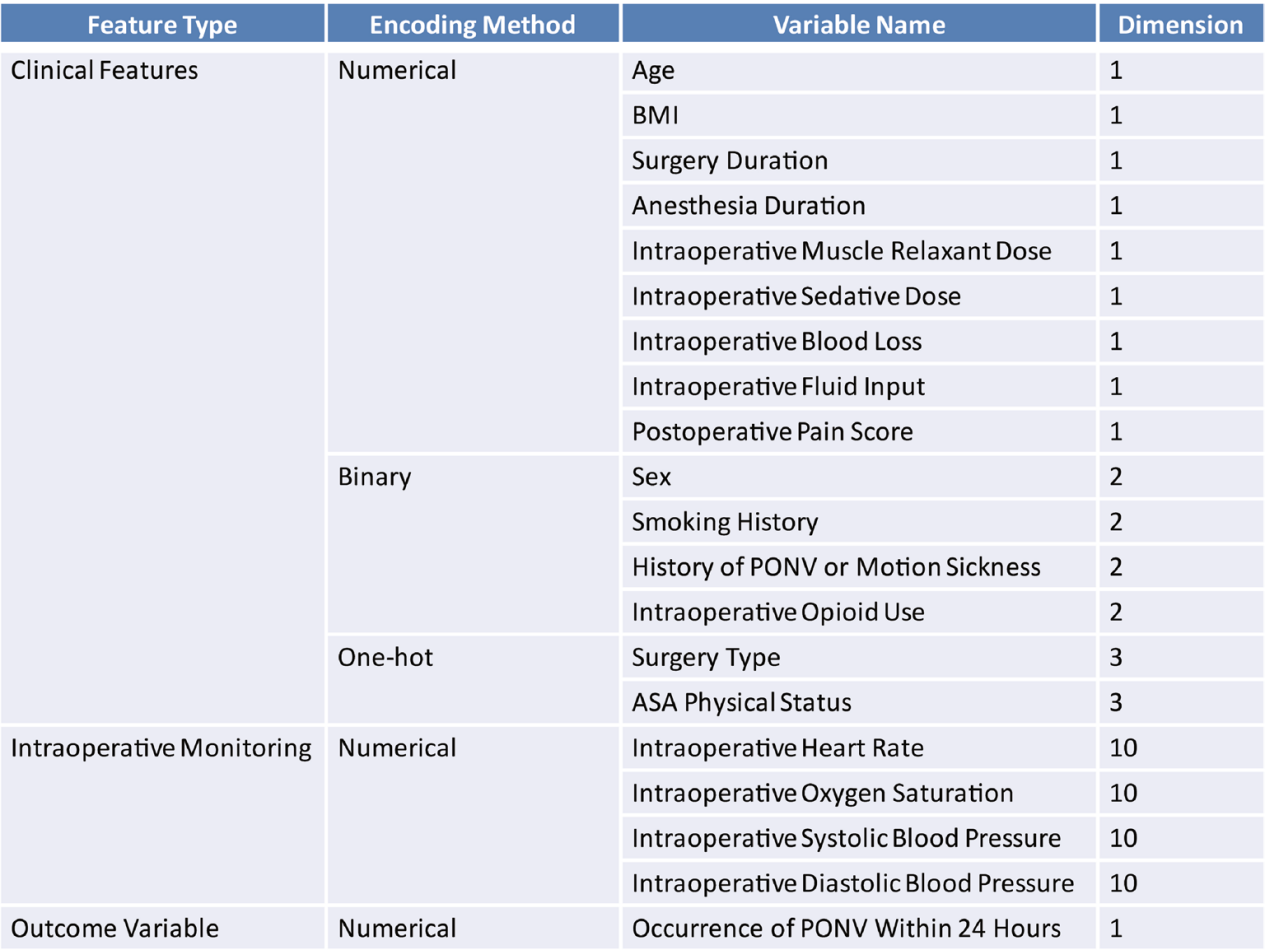
Summary of model variables, encoding strategies, and feature dimensions

### Model development

To accurately model the heterogeneous characteristics and interactions among perioperative variables, we developed a deep learning framework named DSPONVNet. This model was specifically designed to integrate structured clinical variables with intraoperative physiological monitoring data, effectively capturing both nonlinear feature interactions and temporal dynamics.

#### Model architecture

DSPONVNet comprises two parallel subnetworks tailored for heterogeneous input types, followed by a self-attention-based fusion mechanism.

The Multilayer Perceptron (MLP) subnetwork processes structured clinical features. It consists of three hidden layers with 128, 64, and 32 neurons, respectively ,decreasing structure that allows higher-order representations to be extracted while reducing the risk of overfitting. Each layer is followed by a rectified linear unit (ReLU) activation function, which introduces non-linearity and mitigates the vanishing gradient problem, along with batch normalization and dropout with a rate of 0.3 to improve generalization and stabilize training. The Long Short-Term Memory (LSTM) subnetwork handles intraoperative time-series monitoring data, including heart rate, oxygen saturation, systolic blood pressure, and diastolic blood pressure. These signals were recorded every 2 minutes during the final 20 minutes of surgery, yielding 10 time points per variable. The LSTM network contains one hidden layer with 64 units , which was chosen as a balance between modeling temporal patterns effectively and maintaining computational efficiency. This representation is then compressed using average pooling into a fixed-length feature vector. The final hidden layer output of the MLP and the fixed-dimensional representation from the LSTM were concatenated into a single joint feature vector, providing a unified input for the subsequent self-attention module.

**Fig. 2:**
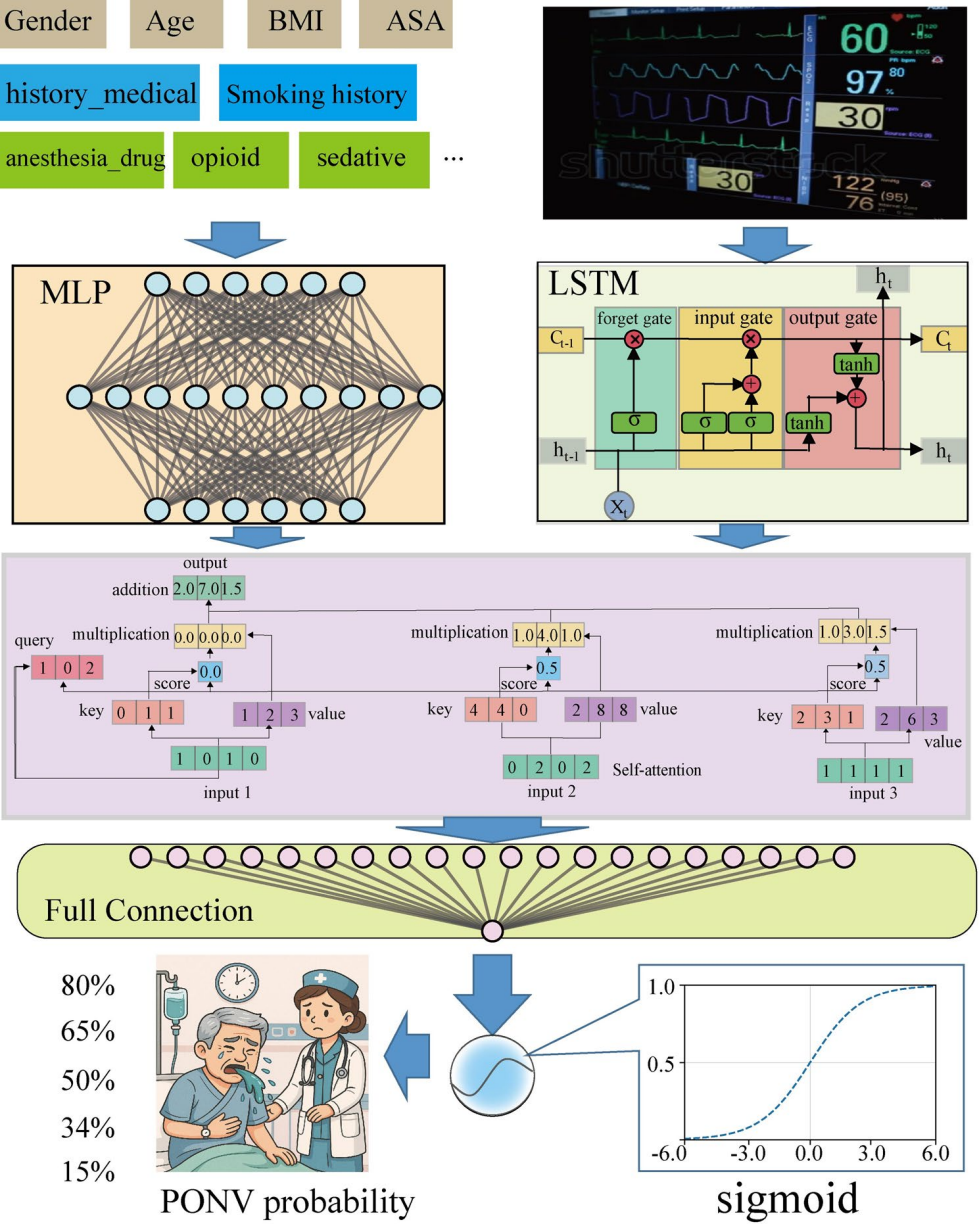
Overall architecture of the proposed DSPONVNet

To integrate multimodal information, DSPONVNet employs a single-layer self-attention mechanism for feature fusion. The attention module computes scaled dot-product attention using 64-dimensional query, key, and value matrices. A residual connection and layer normalization are applied after the attention layer to stabilize training and enhance global feature interactions. The fused representation is then passed through a fully connected output layer with a sigmoid activation function to generate the probability of PONV occurrence. The overall architecture of DSPONVNet is illustrated in Fig. 2

#### Training configuration

**Fig. 3:**
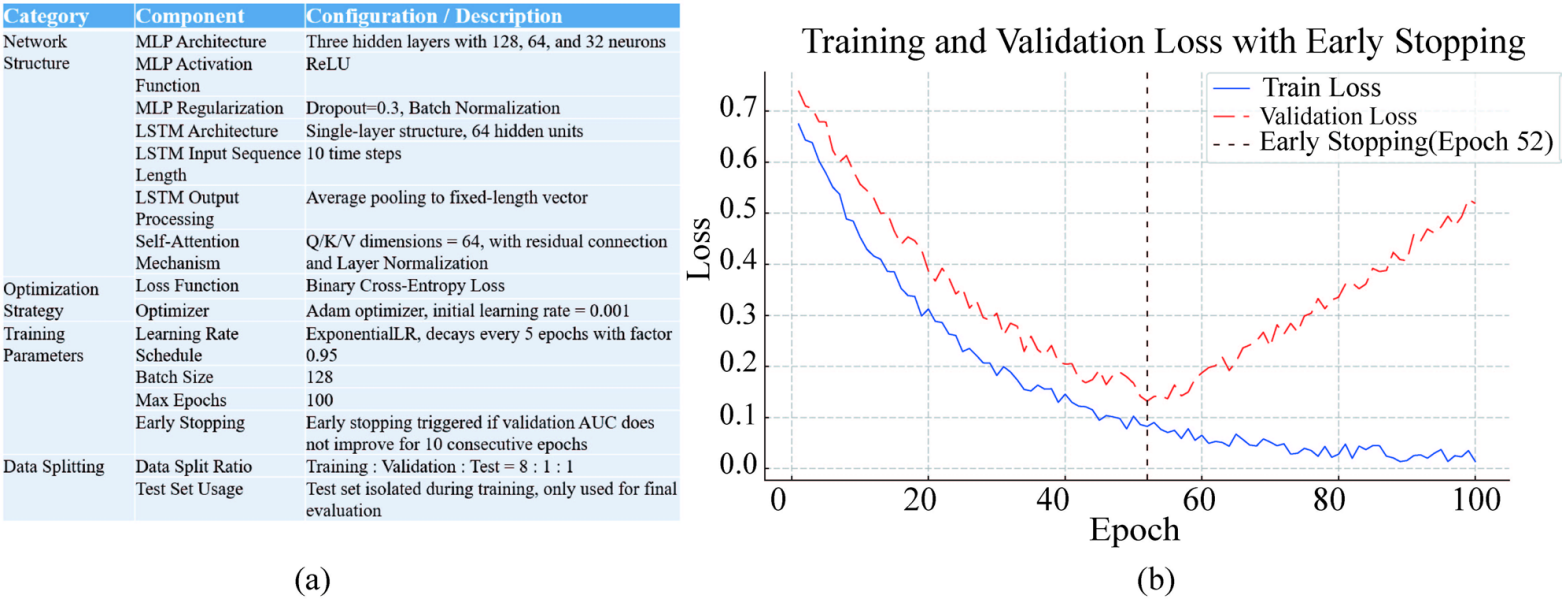
Overview of model configurations and training/validation loss curves. (**a**) DSPONVNet architecture and training configuration, including network architecture, optimizer settings, and data splitting strategy; (**b**) Training and validation loss curves, with early stopping triggered at epoch 53

Model training was performed using PyTorch v1.13.1 on a high-performance computing server equipped with an NVIDIA Tesla P100(16GB) and CUDA 12.2. The binary cross-entropy loss function was used to optimize the model parameters, and the Adam optimizer was employed with an initial learning rate of 0.001. To promote convergence and prevent overfitting, an exponential decay strategy was applied, reducing the learning rate by a factor of 0.95 every 5 epochs. The batch size was set to 128, and the maximum number of training epochs was limited to 100. An early stopping mechanism was implemented to prevent overfitting, which terminated training if the validation AUC did not improve over 10 consecutive epochs. This approach effectively preserved the model’s generalization capability. The dataset was randomly partitioned into training (80%), validation (10%), and testing (10%) subsets. The test set remained strictly isolated throughout the training and hyperparameter tuning processes to ensure an unbiased evaluation of model performance. To further evaluate the robustness of the proposed model, a ten-fold cross-validation procedure was performed. The obtained results were consistent across folds and aligned closely with those derived from the independent test set, indicating stable and reliable model performance. Therefore, only the results on the held-out test set are reported for clarity. The core training configurations and loss trajectories are illustrated in Fig. 3, including detailed network architecture, optimizer settings, and the application of early stopping at epoch 53. During the entire training process, each epoch required approximately 3.8 seconds, and the full training process of 100 epochs took around 382 seconds to complete. During inference, the model exhibited high computational efficiency, generating a prediction for a single patient record within about 1.15 milliseconds under the experimental conditions. In the present study, the network configuration was determined empirically based on prior experience and preliminary tests rather than through exhaustive hyperparameter optimization. While this setup yielded satisfactory predictive performance and stable convergence, further improvements may still be achievable with more systematic tuning. The hyperparameters reported in this study were empirically selected and kept fixed for our dataset, but they may require tuning when applying DSPONVNet to other datasets or clinical contexts.

#### Performance evaluation

To evaluate the predictive performance of DSPONVNet, we compared it with five baseline models widely used in clinical risk prediction: logistic regression, random forest (RF), extreme gradient boosting (XGBoost), LightGBM, and a conventional multilayer perceptron (MLP). All baseline models were implemented using standard Python libraries—Scikit-learn for logistic regression and RF, the official Python APIs for XGBoost and LightGBM, and PyTorch for MLP. Hyperparameters for all models were fine-tuned using grid search to ensure a fair comparison. Each model was trained and evaluated using the same dataset split (training: 80%, validation: 10%, testing: 10%) and identical input features. Classification performance was assessed on the test set using several standard metrics, including the area under the receiver operating characteristic curve (ROC-AUC), area under the precision-recall curve (PR-AUC), F1 score, precision, and recall. These metrics provide complementary perspectives on model discrimination and robustness, particularly under imbalanced class distributions commonly encountered in clinical datasets. All reported model evaluations were conducted exclusively on the independent test set (10% of the dataset), while the training and validation sets were used only for model fitting and hyperparameter tuning.

#### Feature importance analysis

To improve the explainability of DSPONVNet in clinical settings, we employed SHAP (SHapley Additive exPlanations), a widely used model-agnostic algorithm for quantifying feature contributions in machine learning models [19]. Based on cooperative game theory, SHAP assigns each input feature a contribution value for a specific prediction, reflecting its marginal impact under all possible feature combinations. Compared with permutation importance or linear regression coefficients, SHAP provides greater consistency and local accuracy and supports both global and instance-level explanations, making it particularly suitable for complex deep learning models [20–22]. For structured static features (e.g., sex , ASA classification, surgery duration), each SHAP value directly reflects the feature’s marginal effect on the model prediction. For time-series monitoring variables (e.g., heart rate, systolic blood pressure), SHAP values were calculated at each time step across the 10 recorded time points and then averaged to produce a single importance score per variable. This aggregation enabled consistent comparisons between scalar and sequential features, consistent with established time-series SHAP analysis methodologies [23, 24].

The SHAP analysis was conducted using the shap Python package (v0.41.0) in conjunction with the PyTorch implementation of DSPONVNet. We applied the DeepExplainer method to interpret outputs from the multimodal fusion layer, using 100 representative test samples as the background dataset. Global feature importance was ranked based on the mean absolute SHAP values across the test set. Visualization was performed using SHAP’s summary_plot function to generate both global bar charts and swarm plots. These visualizations provided intuitive insights into feature importance rankings, value distributions, and their directional effects on model outputs, facilitating a deeper understanding of the model’s decision-making process.

## Results

This section presents the experimental results of DSPONVNet, including performance comparison with baseline models and SHAP-based feature attribution analysis.

### Evaluation outcomes of model performance

To ensure fair and consistent comparison across all models, each baseline model was independently optimized under identical experimental conditions using standard parameter selection procedures. The hyperparameter search was guided by common practices in machine learning for clinical prediction tasks, focusing on model stability, convergence, and generalization. Table 3 summarizes the selected parameter configurations for each model.

**Table 3:**
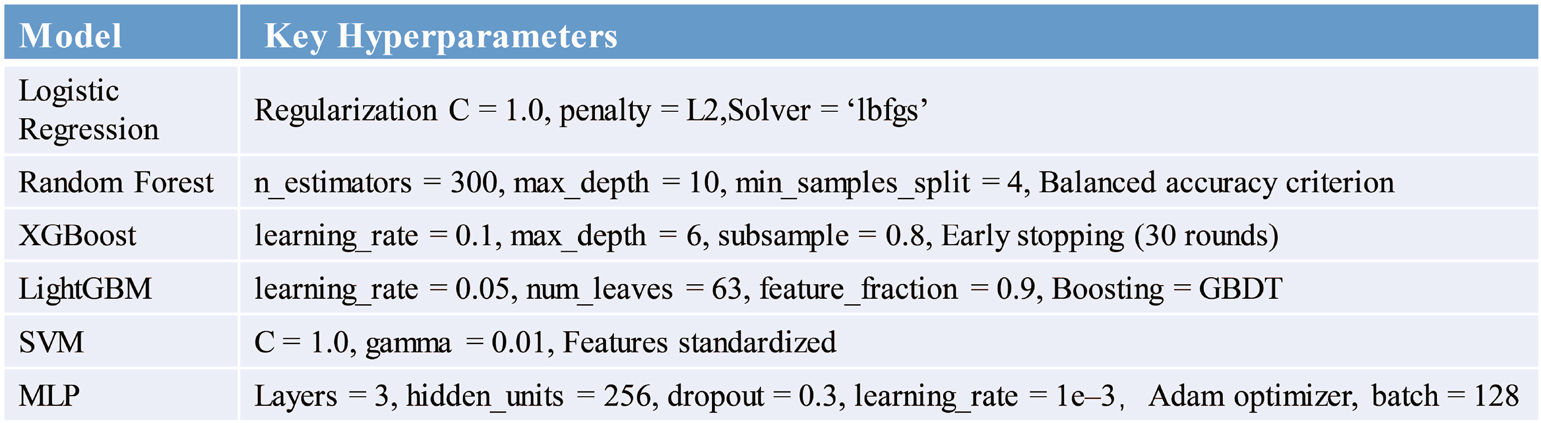
Key hyperparameter configurations of benchmark models

The predictive performance of DSPONVNet, evaluated under the experimental framework described in Training configuration section, is summarized in Table 4 along with the results of the baseline models for comparison. As shown in Table 4, DSPONVNet outperformed all baseline models, achieving a ROC-AUC of 0.9376, an F1 score of 0.8701, and a PR-AUC of 0.9189. Compared with the best-performing baseline model (MLP), DSPONVNet improved the ROC-AUC by 5.6% and the F1 score by 13.4%. In contrast, logistic regression exhibited the poorest performance (ROC-AUC = 0.556), underscoring its limited ability to capture complex nonlinear interactions within the data.

**Table 4:**
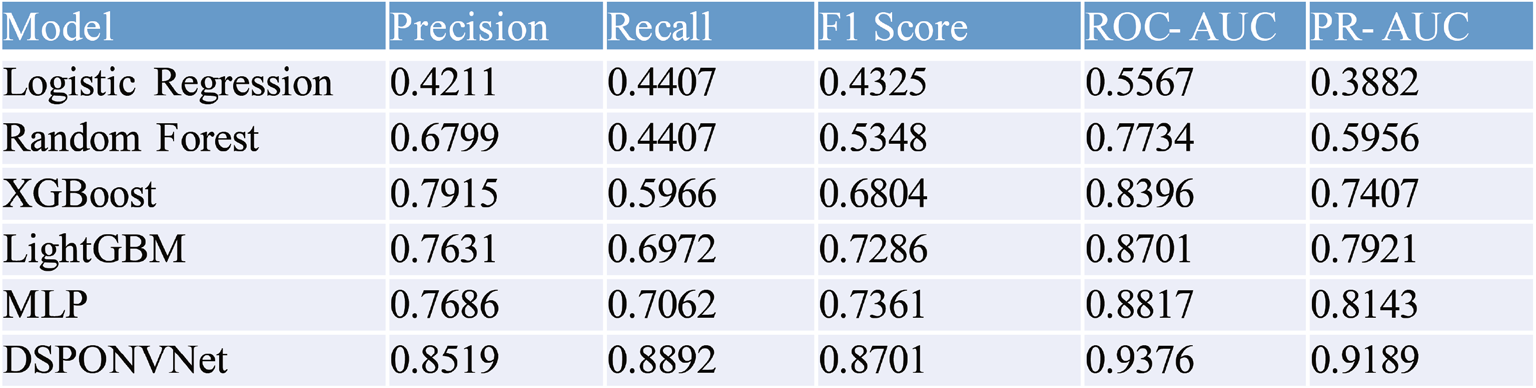
Predictive performance of different models

**Fig. 4:**
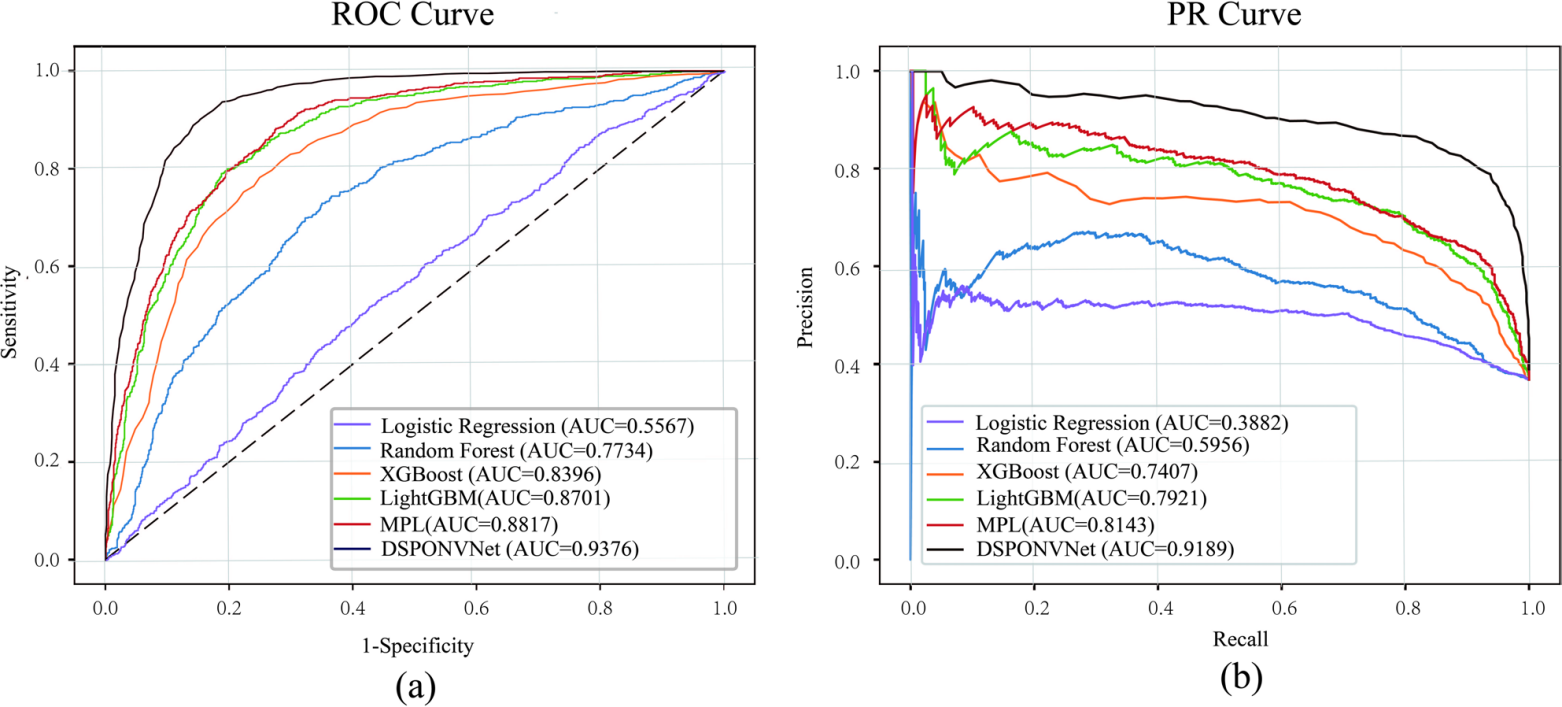
ROC and precision–recall curves for evaluating model performance. (**a**) Receiver operating characteristic (ROC) curves of DSPONVNet and baseline models. (**b**) Precision–recall (PR) curves under class-imbalanced conditions

Figure 4 illustrates the ROC and precision–recall (PR) curves for all models. DSPONVNet demonstrated superior discriminative performance across all thresholds, with its ROC curve closely approaching the top-left corner. The PR curve remained consistently elevated, indicating strong performance in identifying positive (PONV) cases under class-imbalanced conditions.

### Insights into feature importance

**Fig. 5:**
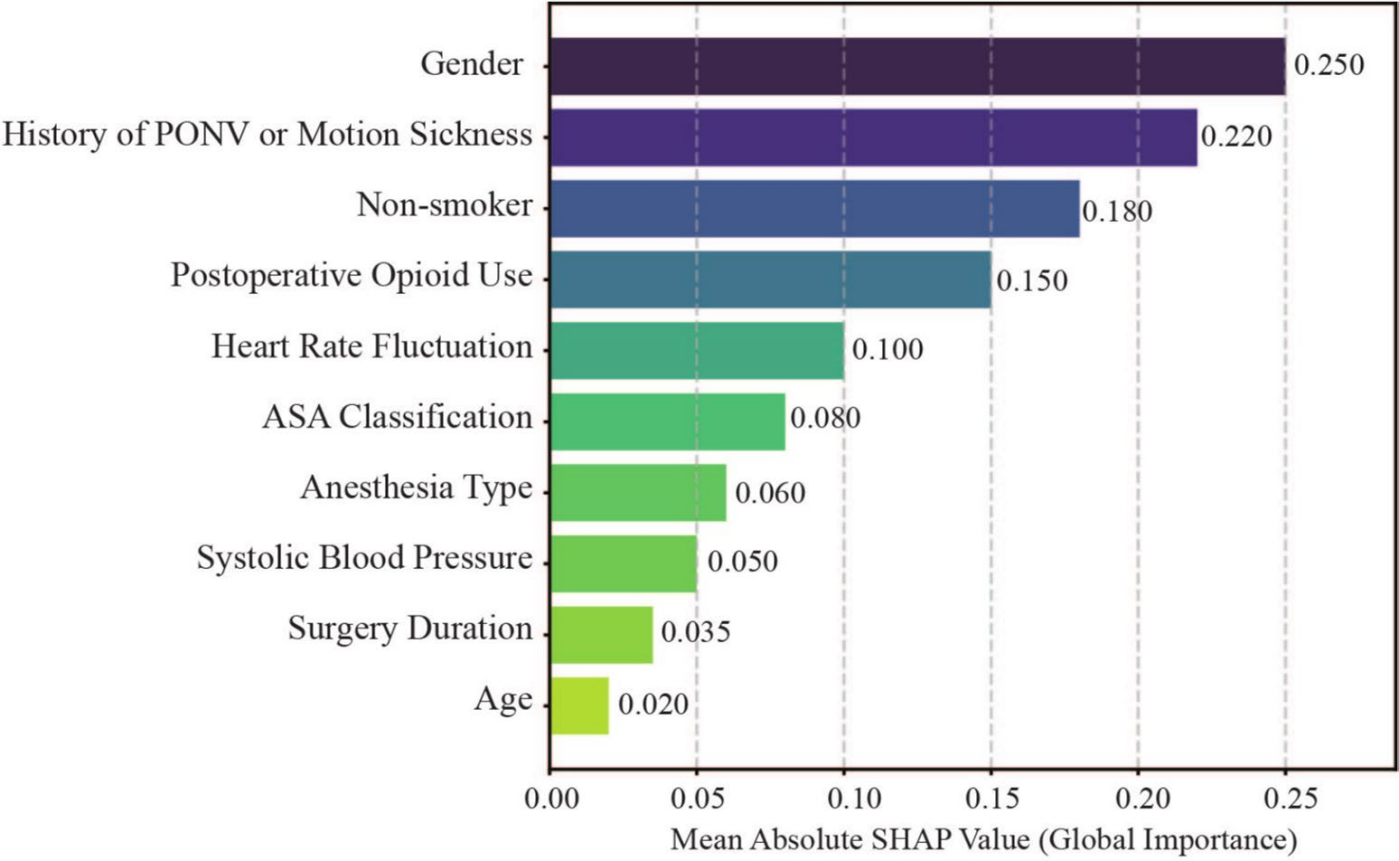
Global feature importance ranking derived from DSPONVNet

To gain insights into the key factors influencing model predictions, we conducted a feature attribution analysis using SHAP. The global feature importance, ranked by the mean absolute SHAP values, is presented in Fig. 5. The top five most influential features were sex, history of PONV or motion sickness, non-smoking status, postoperative opioid use, and heart rate fluctuation.

**Fig. 6:**
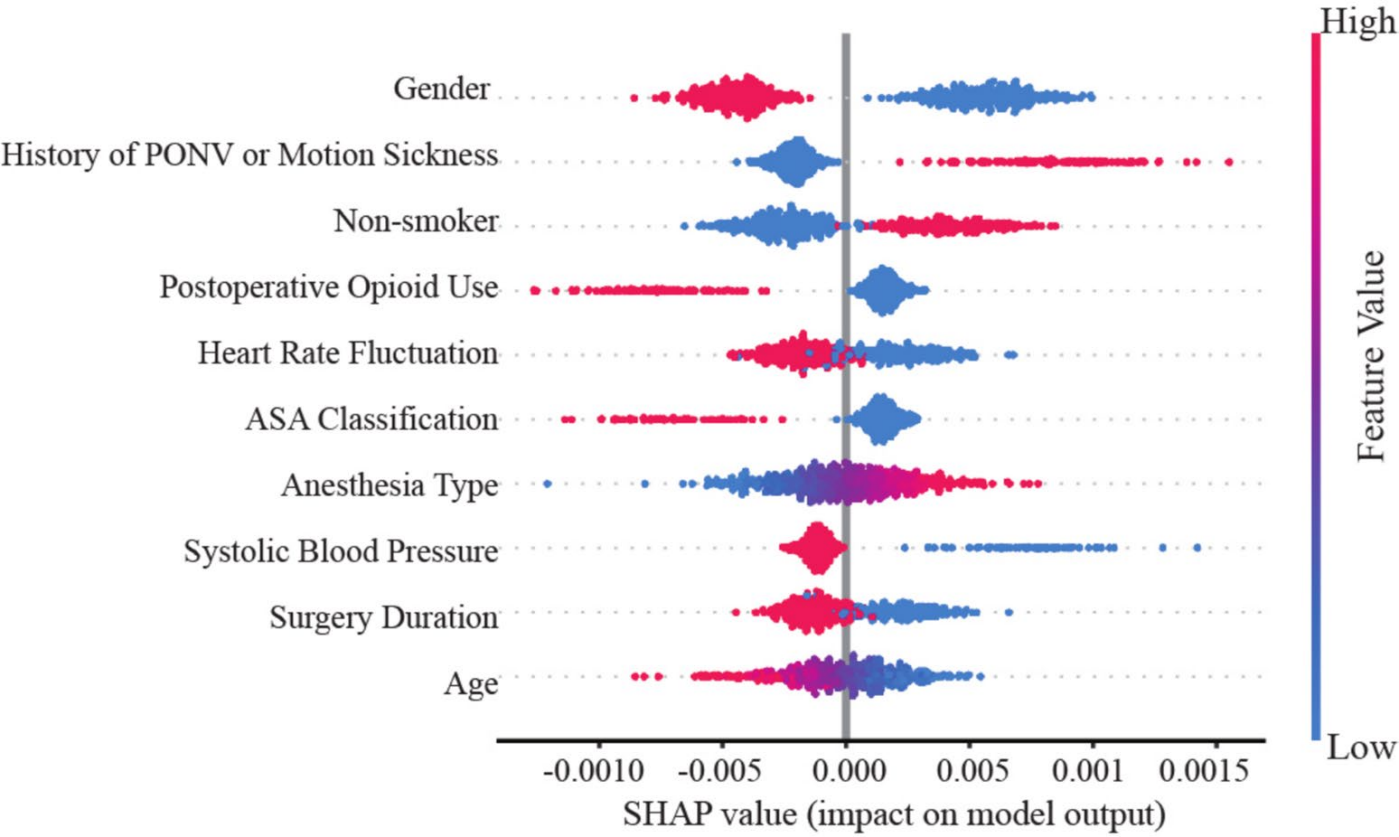
SHAP summary swarm plot of predictive feature contributions

Notably, heart rate fluctuation —a dynamic physiological indicator—ranked among the top five most important predictors, surpassing several traditional static features such as ASA classification and anesthesia type. This finding highlights the added predictive value of intraoperative dynamic monitoring data in PONV risk prediction. Figure 6 presents the SHAP summary plot, illustrating the individual effects of feature values on prediction outcomes. For instance, higher values of heart rate fluctuation and a prior history of PONV corresponded to positive SHAP values, indicating an increased predicted risk. In contrast, male sex and a history of smoking were associated with negative SHAP values, reflecting a lower predicted risk. Overall, most intraoperative features contributed less prominently, which may reflect either the inherently weaker role of dynamic fluctuations in PONV risk or the limitations of the current evaluation approach.

## Discussion

### Key outcomes and comparative performance

DSPONVNet demonstrated significant advantages over conventional machine learning models in predicting PONV risk. While the best-performing baseline model (MLP) achieved moderate predictive results, DSPONVNet notably improved both discrimination and robustness, achieving a ROC-AUC of 0.9376 and an F1 score of 0.8701. These improvements highlight the model’s superior ability to capture complex nonlinear interactions and leverage intraoperative dynamic monitoring data, addressing limitations commonly observed in traditional models such as logistic regression. This performance advantage lays a solid foundation for advancing individualized risk assessment and optimizing perioperative decision-making.

### Feature insights and interpretability

SHAP analysis confirmed that both traditional clinical factors and intraoperative dynamic variables played important roles in PONV risk prediction. While established predictors such as female sex and a history of PONV or motion sickness remained dominant, the emergence of heart rate fluctuation as a key dynamic predictor suggests that intraoperative physiological instability may reflect heightened autonomic nervous system sensitivity or perioperative stress responses. These insights highlight the need to closely monitor intraoperative physiological fluctuations and consider them in real-time risk assessments, potentially informing more targeted perioperative interventions. It is worth noting that in this study, SHAP was mainly applied to interpret model predictions and provide clinical insights. Future research will further examine its potential for feature selection and model refinement.

### Clinical applicability and limitations

By integrating dynamic intraoperative monitoring data, DSPONVNet provides not only improved predictive accuracy but also clinically meaningful interpretability, offering practical value for perioperative management. The model leverages real-time physiological fluctuations—particularly heart rate variability—to capture subtle intraoperative risk signals that are often overlooked by static clinical assessments. The model’s predictions can also inform postoperative nursing strategies by identifying patients at elevated risk, facilitating targeted monitoring and timely interventions. Furthermore, the SHAP-based analysis provides additional interpretive value by illustrating the relative contribution of key predictors to the model’s output, offering clinicians clearer insight into factors associated with PONV risk.

Despite these advantages, this study has certain limitations. First, the model was developed and validated using data from a single center, potentially limiting its generalizability to broader clinical populations. Second, the current analysis primarily focused on global feature importance, while detailed temporal patterns within dynamic monitoring data remain underexplored. Thirdly, missing continuous variables (e.g., BMI, surgery duration) were imputed using population means. This approach, although simple and commonly used, does not incorporate missing-data indicators and therefore may overlook potential information encoded in the missingness pattern, which could introduce bias in certain settings. Finally, a data-availability–related limitation should be noted: a small proportion of cases (< 1%) were excluded because the PONV outcome could not be assessed due to postoperative events, with no expected serious impact on the core results.

Future studies should address these limitations by incorporating multicenter datasets, applying advanced temporal modeling techniques, and adopting more robust missing-data handling strategies to further enhance model performance and clinical applicability.

## Conclusions

This study proposed DSPONVNet, a multimodal deep learning framework that integrates structured clinical features with dynamic intraoperative monitoring data for PONV risk prediction. By effectively capturing complex temporal dependencies and nonlinear interactions, DSPONVNet achieved superior predictive performance and enhanced interpretability compared to traditional models. These findings demonstrate the clinical value of incorporating real-time physiological monitoring data into perioperative risk assessment, offering a novel and data-driven approach to support individualized patient management and optimize perioperative care strategies. Future research will focus on external validation across diverse clinical environments to assess the model’s generalizability. In addition, exploring advanced temporal modeling and interpretability techniques will be critical to further improving predictive accuracy and ensuring broader clinical applicability.

## Data Availability

The datasets generated and analyzed during the current study are not publicly available due to institutional regulations and privacy protection policies of the First Hospital of Jilin University. Access to the datasets requires prior approval from the hospital’s data governance board. Requests to access the data should be directed to the corresponding authors: Z. X.(xuezp@jlu.edu.cn) or Y.L. (yliu66@jlu.edu.cn).
